# C-C Motif Chemokine Ligand-17 as a Novel Biomarker and Regulator of Epithelial Mesenchymal Transition in Renal Fibrogenesis

**DOI:** 10.3390/cells10123345

**Published:** 2021-11-29

**Authors:** Yi-Hsien Hsieh, Wen-Chien Wang, Tung-Wei Hung, Chu-Che Lee, Jen-Pi Tsai

**Affiliations:** 1 Institute of Medicine, Chung Shan Medical University, Taichung 40201, Taiwan; hyhsien@csmu.edu.tw (Y.-H.H.); davidwang19950526@gmail.com (W.-C.W.); 2 Department of Medical Research, Chung Shan Medical University Hospital, Taichung 40201, Taiwan; 3 School of Medicine, Chung Shan Medical University, Taichung 40201, Taiwan; a6152000@ms34.hinet.net; 4 Department of Medical Education, Taichung Vererans General Hospital, Taichung 40705, Taiwan; 5 Division of Nephrology, Department of Medicine, Chung Shan Medical University Hospital, Taichung 40201, Taiwan; 6 Department of Medicine Research, Dalin Tzu Chi Hospital, Buddhist Tzu Chi Medical Foundation, Chiayi 62247, Taiwan; dm731849@tzuchi.com.tw; 7 School of Medicine, Tzu Chi University, Hualien 97004, Taiwan; 8 Division of Nephrology, Department of Internal Medicine, Dalin Tzu Chi Hospital, Buddhist Tzu Chi Medical Foundation, Chiayi 62247, Taiwan

**Keywords:** chronic kidney disease, epithelial mesenchymal transition, CCL17, unilateral urethral obstruction

## Abstract

CCL17, a chemotactic cytokine produced by macrophages, is known to promote inflammatory and fibrotic effects in multiple organs, but its role in mediating renal fibrosis is unclear. In our study cohort of 234 chronic kidney disease (CKD) patients and 65 healthy controls, human cytokine array analysis revealed elevated CCL17 expression in CKD that correlated negatively with renal function. The area under the receiver operating characteristic curve of CCL17 to predict the development of CKD stages 3b–5 was 0.644 (*p* < 0.001), with the optimal cut-off value of 415.3 ng/mL. In vitro over-expression of CCL17 in HK2 cells had no effect on cell viability, but increased cell motility and the expression of α-SMA, vimentin and collagen I, as shown by western blot analysis. In a unilateral ureteral obstruction (UUO) mouse model, we observed significantly increased interstitial fibrosis and renal tubule dilatation by Masson’s Trichrome and H&E staining, and markedly increased expression of CCL17, vimentin, collagen I, and α-SMA by IHC stain, qRTPCR, and western blotting. CCL17 induced renal fibrosis by promoting the epithelial-mesenchymal transition, resulting in ECM accumulation. CCL17 may be a useful biomarker for predicting the development of advanced CKD.

## 1. Introduction

The progression of chronic kidney disease (CKD) is a complex, multi-stage process that ultimately leads to the destruction of the normal renal structure. Regardless of the nature of the initial injuries, renal fibrosis is the final pathway leading to end-stage renal disease [1]. The final outcome of renal fibrosis depends on the balance between healing and scarring. Despite strategies to control modifiable risk factors for CKD progression, such as diabetes mellitus, hypertension, proteinuria, renin-angiotensin system blockade, and nephrotoxin exposure [2,3], the prevalence of CKD is increasing. Thus, the search for a novel and effective therapy to inhibit the key mediators of renal scarring is warranted. 

Chronic sustained kidney damage leads to trans-differentiation of renal tubular epithelial cells, known as the epithelial–mesenchymal transition (EMT), eventually resulting in apoptosis with the deposition of collagenous extracellular matrix (ECM) [4]. During the EMT, kidney tubule cells lose their epithelial characteristics and develop the mesenchymal phenotype. This process is mediated by inflammation, as evidenced by studies showing that the extent of inflammatory infiltration correlates with the severity of renal dysfunction and CKD progression [5,6]. Non-resolving tissue injury inevitably perpetuates a vicious cycle of ongoing inflammation, tissue damage, and fibrosis. ECM is deposited by myofibroblasts, which synthesize and store interstitial ECM components, such as collagen type I and III, and fibronectin during wound healing and at scar and fibrosis sites [5,6].

Chemokines are chemo-attractants for specific subsets of inflammatory cells [7,8]. A variety of chemokines and their receptors are associated with fibrogenesis. For example, CXCL16 deficiency has been shown to impair aggregation and myofibroblast production of bone-marrow–derived fibroblasts in the kidney [9,10], while CCR2 deficiency significantly reduces the production of bone-marrow derived myofibroblasts [11]. In a murine model, macrophages were observed to activate collagen production by myofibroblasts via modulation of thymus activation-regulated chemokine (CCL17) expression through the interaction of CD248 and galectin-3 [12]. CCL17 is elevated in bronchoalveolar lavage from idiopathic pulmonary fibrosis [13,14] and positively correlates with disease activity in atopic dermatitis [15]. CCL17 further modulates the severity of peritoneal function via promoting macrophage transformation to the pro-fibrotic phenotype and activating peritoneal fibroblasts to induce peritoneal fibrosis [16]. Renal allograft biopsies show significant upregulation of chemokine genes, including CCL17, between subclinical inflammation and clinical inflammation [17]. In CKD patients, the concentrations of serum and urinary chemokines are elevated in advanced-stage, compared to early-stage CKD [18]. The roles of CCL17 and CCL22 in the progression of renal fibrosis remain unclear. 

Based on these previous findings, we hypothesized that sustained inflammation or renal injury stimulates local and systemic chemokine secretion and the recruitment of inflammatory cells that contribute to the progression of renal fibrosis, resulting in end-stage renal disease. This study aims to elucidate the mechanism underlying CKD-related renal fibrosis and to discover possible novel biomarkers to aid in the diagnosis of CKD. 

## 2. Materials and Methods

### 2.1. Participants and Biochemical Analysis

From April 2015 to November 2017, we recruited 65 normal subjects and 234 CKD patients from a local hospital in Southern Taiwan. Fasting blood samples (approximately 5 mL) were immediately centrifuged, and serum levels of blood urea nitrogen and creatinine were determined using an auto-analyzer (Siemens Dimension RxL Max, Siemens Healthcare GmbH, Henkestr, Germany). Serum levels of CCL17 and CCL22 were measured using commercially available enzyme-linked immunosorbent assays (R&D Systems, Inc. Minneapolis, MN, USA). The Modification of Diet in Renal Disease (MDRD) and Chronic Kidney Disease Epidemiology Collaboration (CKD-EPI) were determined as follows: eGFR_MDRD_ = 186 × Scr^−1.154^ × age^−0.203^ × 0.742 (if female) × 1.212 (if black patient); eGFR _CKD-EPI_ = 141 × min (S_Cr_/κ, 1)^α^ × max (S_Cr_/κ, 1)^−1.209^ × 0.993^Age^ × 1.018 [if female] × 1.159 [if black patient]. These values were applied to the calculation of the estimated glomerular filtration rate (eGFR). Patients were classified as CKD stages 1, 2, 3a, 3b, 4, and 5 according to the Kidney Disease: Improving Global Outcomes (KDIGO) guidelines [19]. All patients were further categorized into groups as normal population, early CKD (stages 1 to 3a), and advanced CKD (stages 3b to 5). The Protection of the Human Subjects Institutional Review Board of Dalin Tzu-Chi approved this study (IRB B10501007). All participants provided their informed consent before participating in this study.

### 2.2. Chemical Reagent and Antibody

MTT (3-(4,5-dimethylthiazol-2-yl)-2,5-diphenyltetrazolium bromide), DAPI solution (1 mg/mL), DMSO (Dimethyl sulfoxide, for molecular biology), and Tris-HCl (Tris hydrochloride, purify > 99%) were purchased from Merck (Darmstadt, Germany). Immunoblotting antibodies against GFP (2956S) were obtained from Cell Signaling Technology (Beverly, MA, USA). Antibody against vimentin (3634-100) was obtained from BioVision, (Milpitas, CA, USA). Antibodies against α-SMA (sc-53015), collagen I (sc-59772), GAPDH (sc-365062), HRP-mouse IgG and HRP-rabbit IgG were purchased from Santa Cruz Biotechnology (Santa Cruz, CA, USA). Antibody against human CCL-17 (AF364) was purchased from R&D Systems, Inc (Minneapolis, MN, USA). ECL detection reagent was purchased from EMD Millipore (Burlington, MA, USA). The design and synthesis of GFP-CCL17 was performed by AllBio company (Taipei, Taiwan).

### 2.3. Unilateral Ureteral Obstruction (UUO) and Immunohistochemistry

The animal experiment protocol was approved by the Institutional Animal Care and Use Committee of Chung Shan Medical University, Taichung, Taiwan (IACUC number: 2230). Six-week-old C57BL/6 mice (weight, 20–23 g) were purchased from National Laboratory Animal Center (Taipei City, Taiwan) and kept in a light- and humidity-regulated environment (12 h light/12 h dark cycle) at 23 °C. The mice were free to feed and drink sterilized water. Unilateral ureteral obstruction (UUO) was performed in the rodents as previously reported [20]. Under isoflurane anesthesia, the mice were placed in the lateral decubitus position. The fur over the flank area was shaved using an electric shaver followed by sterilization with iodine tincture. In a lateral-posterior approach, a 3–4 cm incision was made parallel to the spine. The soft tissue was gently dissected until the kidney, proximal ureter, and distal ureter were clearly visible. The proximal and distal ureters were ligated using two surgical ties with nylon sutures. After ligation, the kidney, ureter, and soft tissue were put back into their original positions. The wound was then closed using nylon sutures. After 7 or 14 days, the mice were sacrificed and the kidney tissues were photographed. The kidney tissues were coated on glass slides for overnight, then these kidney tissues were treated with a primary antibody against collagen I (1:200), α-SMA (1:100), and vimentin (1:200) in a detected buffer and then added with secondary antibodies for 1 h. The interstitial collagen fibers were measured with Masson’s Trichrome staining.

### 2.4. Human Cytokine Assay

The Proteome Profiler Human XL Cytokine Array Kit (ARY022B, R&D, Minneapolis, MN, USA), including the 105 human cytokines were measured. Plasme was collected from the normal and different stages of CKD patients and centrifuged for 15 min, according to the manufacturer’s instructions. Profiles of mean spot pixel density were created using a transmission-mode scanner and image analysis software.

### 2.5. Cell Culture

The human proximal tubule cell line HK2 was purchased from the Food Industry Research and Development Institute. HK2 cells were cultured in DMEM-F12 culture medium supplemented with FBS (Fetal Bovine Serum) at 37 °C in humidified air with 5% CO_2_.

### 2.6. Cell Cycle Assay

The cell cycle assay was performed as previously described [21]. Cells were treated with PI reagent, incubated for 30 min at room temperature, fixed in ice-cold 70% alcohol at −20 °C for 2 days, and afterwards the alcohol was removed by washing twice in PBS. A Muse Cell Analyzer (Millipore) was used to determine the cell cycle phase of the treated cells.

### 2.7. DNA Transient Transfection

Briefly, HK2 cells (5 × 10^4^) were seeded into 6 cm culture dishes for 24 h and then mixed with RNAiMAX reagent (Thermo Fisher Scientific, Waltham, MA, USA) and GFP or GFP-CCL17 plasmid in a serum-free medium for 6 h. Fresh medium (20% FBS) was added, and the cells were incubated for another 42 h. Western blotting and qRTPCR were used to determine the transfection efficiency.

### 2.8. Assessment of Cell Growth by MTT Assay

GFP or GFP-CCL17 cells (3 × 10^4^ cells/well) were seeded into 24 well plates for 24, 48, or 72 h. MTT reagent was added to the cells at the indicated times followed by a 30-min incubation. The medium then was removed and isopropanol was added. The absorbance at 405 nm was determined using a Multiskan MS ELISA reader (Labsystems, Helsinki, Finland).

### 2.9. Assessment of Gene Expression by qRT-PCR

Total RNA was extracted from UUO tissue and transfected cells using the TRIzol RNA Isolation Reagent (Thermo Fisher Scientific, Waltham, MA, USA) according to the manufacturer’s instructions. Chloroform was added and the solution and was mixed for 10 min. The supernatant was removed and placed in a new tube, to which 500 uL isopropanol was added. After sitting on ice for 20 min, the total RNA purity and content were measured at 260–280 nm using the Nanodrop spectrophotometer (Thermo Fisher Scientific, Waltham, MA USA). cDNA was synthesized from the RNA using the GoScript Reverse Transcription Mix (Promega Corporation, Madison, WI, USA). The template cDNA and GoTaq qPCR Master Mix (Promega, MW, USA) were measured using the ABI PRISM 7700 real-time PCR system (Applied Biosystems, Foster City, CA, USA). The specific mice primer sequences used for qRT-PCR were as follows: mCCL17, Forward: 5′- GTACCATGAGGTCACTTCAGA-3′, Reverse: 5′-CCTTCTTCACATGTTTGTCTTT-3′; mVimentin, Forward: 5′-GAGAACTTTGCCGTTGAAGC-3′, Reverse: 5′-GCTTCCTGTAGGTGGCAATC-3′; mα-SMA, Forward: 5′-GAGGCACCACTGAACCCTAA-3′, Reverse: 5′-CATCTCCAGAGTCCAGCACA-3; mGAPDH, Forward: 5′-GTGCATGAAGGACAGCCTCT-3′, Reverse: 5′-CCACCTTAAAATCTGCAGGC-3. The specific human primer sequences were as follows: hCCL17, Forward: 5′-GGGAGTGCTGCCTGGAGTA-3′, Reverse: 5′-TCTCTTGTTGTTGGGGTCCG-3′; hCollagen I, Forward: 5′-TGAGAGAGGGGTTGTTGGAC-3′, Reverse: 5′-AGGTTCACCCTTCACACCTG-3′; hα-SMA, Forward: 5′-CTATGCCTCTGGACGCACAACT-3′, Reverse: 5′-CAGATCCAGACGCATGATGGCA-3′; hVimentin, Forward: 5′-AGGAAATGGCTCGTCACCTTCGTGAATA-3′, Reverse: 5′-AGGAGTTCGGTTGTTAAGAACTAGAGC-3′; hGAPDH, Forward: 5′-CATCATCCCTGCCTCTACTG-3′, Reverse: 5′- GCCTGCTTCACCACCTTC-3′. GAPDH as a mRNA loading control. The mRNA results of qRTPCR by using the ∆∆Ct method.

### 2.10. In Vitro Migration Assay

GFP- and GFP-CCL17 cells (3 × 10^5^ cells/ chamber) in serum-free DMEM were seeded into the upper wells of a Boyden chamber assay. To the bottom wells was added a DMEM medium (20% FBS), followed by incubation in 5% CO_2_ for 48 h. Cells in the upper chamber were fixed in 100% methanol and stained with 0.1% crystal violet. The cells were observed and counted under a microscope in five fields and photographed at 200× magnification.

### 2.11. Western Blotting

The expression of EMT-related proteins and CCL17 protein was assessed using western blotting as previously described [22]. The GFP- or GFP-CCL17 cells (5 × 10^5^) were maintained in 6 cm culture dishes until they reached 70% confluence and were then lysed in lysis buffer on ice for 15 min. The total proteins (20 mg total) were separated, electrophoresed, transferred to PVDF membranes, and immunoblotted. Primary antibodies against GFP (1:5000), α-SMA (1:1000), vimentin (1:1000), collagen I (1:500), and GAPDH (1:5000) were used for immunoblotting, and the signal was detected using Immobilon HRP Substrate (Millipore, MA, USA). Immunoblot bands were detected using the ImageQuant LAS-4000 mini Analyzer (GE Healthcare, Marlborough, MA, USA).

### 2.12. Statistical Analysis

Clinical variables ware expressed as the mean ± standard deviation, and comparisons between the normal population, early, and advanced CKD groups were analyzed using analysis of variance. Linear correlation analysis was used to determine the relationship between eGFR and CCL17 or CCL22. The receiver operating characteristic (ROC) curve was used to calculate the area under the curve (AUC) to identify the most proper cutoff value of CCL17 and CCL22 for predicting advanced CKD. MedCalc Statistical Software (version 12.7.2; MedCalc Software Bvba, Ostend, Belgium) was used for data analysis. Statistically significant was considered to be *p* < 0.05. All experiment data were analyzed using GraphPad 4.0 software. Student’s t-tests were used to assess the differences between the control and experiment groups. * *p* < 0. 05; ** *p* < 0.01, significant difference.

## 3. Results

### 3.1. Upregulation of CCL17 in Patients with Advanced Chronic Kidney Disease

**Figure 1:**
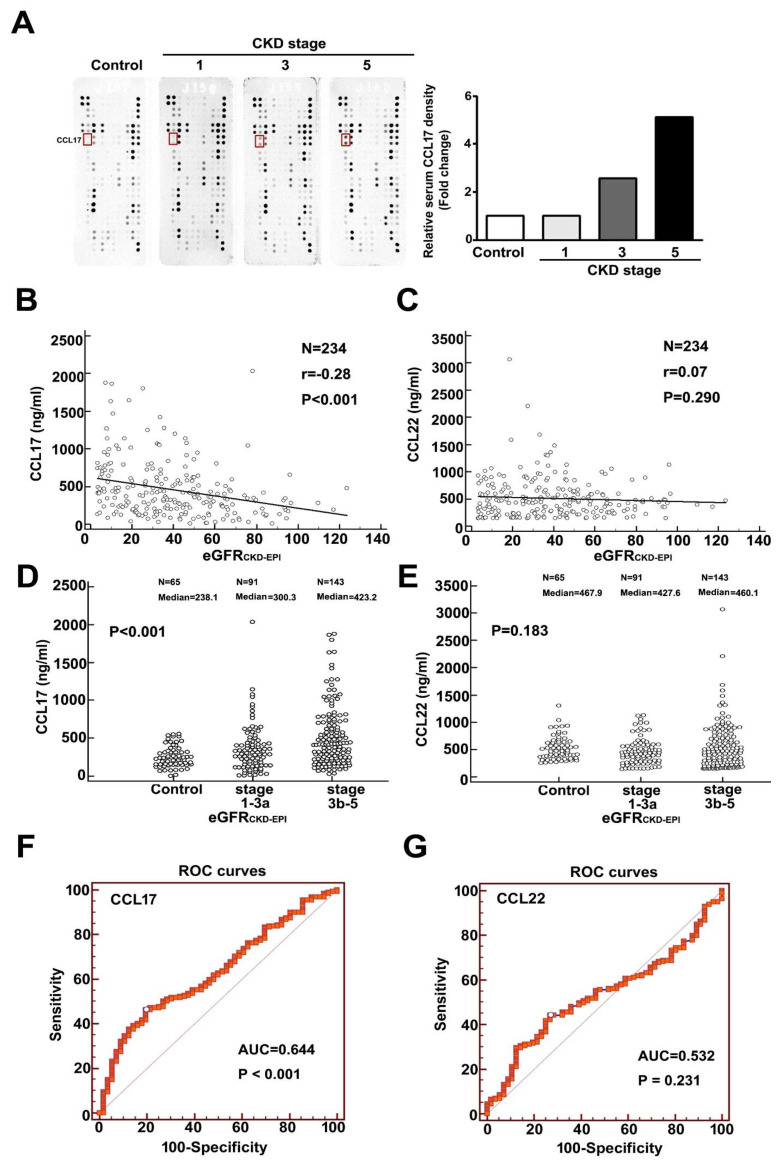
Increasing expression of CCL17 with decreasing renal function. (**A**) Human cytokine array profile of cell lysates from the normal population and patients with CKD stages 1, 3, and 5. CCL17 spots are marked. Correlation analysis of (**B**) CCL17 (**C**) or CCL22 with eGFR_CKD-EPI_ for all CKD patients. Comparison of mean serum values of (**D**) CCL17 (**E**) and CCL22 between the normal population (control), CKD stages 1–3a and stages 3b–5. The area under the receiver operating characteristic curve indicated the diagnostic power of (**F**) CCL17 (**G**) and CCL22 for predicting advanced CKD (stages 3b–5).

We observed upregulated expression of CCL17 as renal function deteriorated (Figure 1A). Table 1 showed the baseline characteristics of eGFR_CKD-EPI_ for normal population and CKD patients. Comparison of the normal population, and CKD stages 1, 3, and 5 revealed a significant progressive increase in CCL17 levels with disease progression (Figure 1A). In addition, a significant negative relationship between CCL17 and eGFR_CKD-EPI_ was observed for all CKD patients (Figure 1B), and serum CCL17 was significantly higher in advanced CKD patients (stages 3b–5) than in early CKD patients (stages 1–3a) and normal groups (Figure 1D). We observed no significant relationship between CCL22 and eGFR_CKD-EPI_ (Figure 1C) in the normal population, early, or advanced CKD patients (Figure 1E). Similar results were observed with the relationship between CCL17 or CCL22 and eGFR_MDRD_ for all CKD patients (Figure S1A,B) and in the normal population (control), stages 1–3a, and stages 3b–5 of CKD patients (Figure S1C,D). Table S1 showed the baseline characteristic of eGFR_MDRD_ for normal population and CKD patients. The area under the receiver operating characteristic curve for CCL17 and CCL22 for predicting the development of advanced CKD was 0.644 (*p* < 0.001) (Figure 1F) and 0.532 (*p* = 0.231) (Figure 1G), respectively. The optimal CCL17 cut-off value for predicting advanced CKD was 415.3 ng/mL, with 53.73% sensitivity and 73% specificity.

### 3.2. Overexpression of CCL17 in HK2 Cells Does Not Affect Their Viability or Cell Cycle Phase Distribution

**Figure 2:**
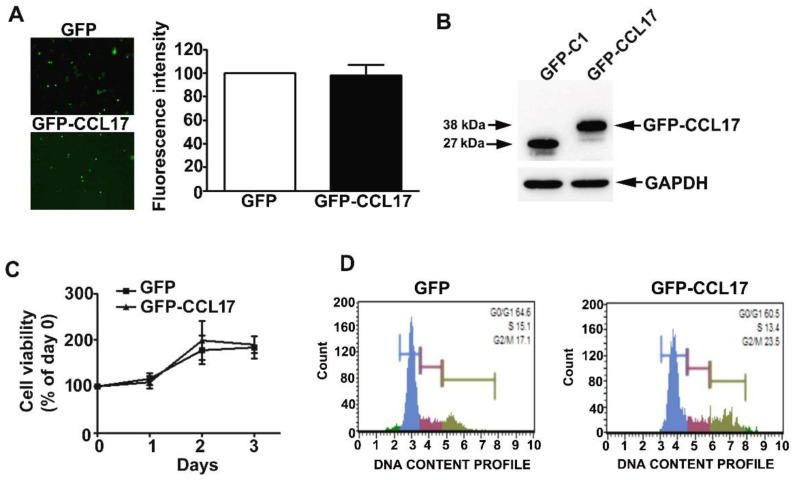
Effects of overexpression of CCL17 on the viability and proliferation of HK2 cells. GFP- or GFP-CCL17 plasmids were transfected into HK2 cells for 48 h. (**A**) Transfection efficiency as determined using fluorescence microscopy. Data are expressed as a percentage of the fluorescence intensity as compared with the GFP cells (**B**) CCL17 protein expression was detected with GFP antibody by western blotting, relative to GAPDH as the loading control; (**C**) cell growth as assessed by MTT cell viability assay; (**D**) cell cycle distribution as assessed by PI staining and flow cytometry. Results are expressed as the mean ± SD of at least three independent experiments.

HK2 cells were effectively transfected with GFP and GFP-CCL17 plasmids, resulting in CCL17 overexpression (Figure 2A,B). No time-dependent effects on cell growth or cell cycle were observed with CCL17 overexpression in HK2 cells, as shown by MTT assay and flow cytometry, respectively (Figure 2C,D).

### 3.3. CCL17 Overexpression Increased HK2 Cell Migration Ability

**Figure 3:**
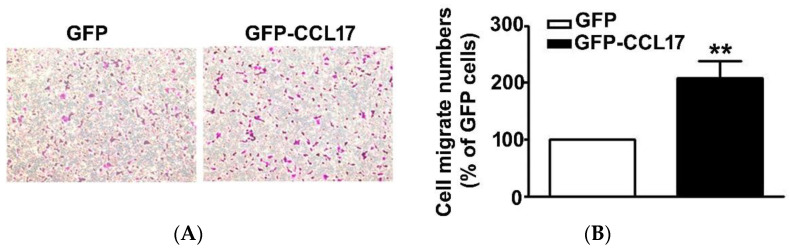
The motility of CCL17-transfected HK2 cells. (**A**) The migration of HK2 cells transfected with GFP- and GFP-CCL17 were determined by using an in vitro migration assay. Cells in the lower surface of the Boyden chamber were stained and photographed under a light microscope at ×400 magnification. (**B**)The number of cells that migrated are quantified and shown as a histogram chart in the right panel. Results are expressed as mean ± SD of at least three independent cell experiments. ** *p* < 0.01 compared with GFP cells.

Since CCL17, overexpression had no effect on the viability of HK2 cells. As such, we further investigated its effect on the migration of HK2 cells using an in vitro migration assay. First was the transfection of GFP or GFP-CCL17 into HK2 cells at 6 cm culture dish for 48 h, after which they were collected and counted, and then the migrate cell number of GFP or GFP-CCL17-HK2 cells by in vitro migration assay were detected. We observed a marked increase in the cell number of GFP-CCL17 cells as compared to GFP cells (Figure 3). These results demonstrated that CCL17 overexpression promoted HK2 cell migration, independent of cell growth.

### 3.4. Overexpressed CCL17 Involved in Epithelial–Mesenchymal Transition (EMT) and Fibrosis in HK2 Cells

**Figure 4:**
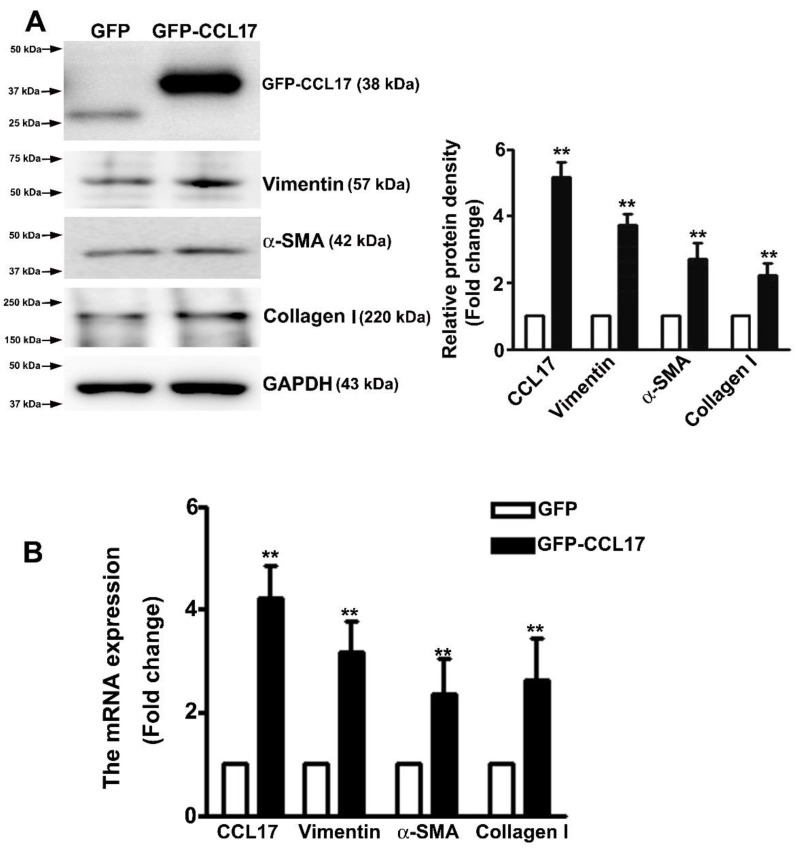
CCL17 regulates EMT-related protein and mRNA expression in HK2 cells; (**A**) Western blot analysis of total HK2 cell lysates to determine the protein expression of CCL17, collagen I, α-SMA, and vimentin. The relative protein density is shown as histograms in the right panel. GAPDH was used to normalize protein loading; (**B**) qRTPCR assay of mRNA expression of CCL17, collagen I, α-SMA, and vimentin. GAPDH was used to normalize mRNA loading. Results are expressed as the mean ± SD of at least three independent experiments. ** *p* < 0.01 compared with GFP cells.

Studies have suggested that the occurrence of EMT could affect the cell migration in renal fibrosis procedure [23]. Western blot analysis was carried out to determine whether CCL17 affected EMT- or fibrotic-related proteins expression in HK2 cells. We observed an increased expression of CCL17, collagen I, α-SMA, and vimentin in overexpressing GFP-CCL17 cells as compared to GFP cells (Figure 4A). Consistent with the qRT-PCR results (Figure 4B), these results indicated that CCL17 overexpression significantly increased the motility of HK2 cells through the regulation of the EMT process. Similar results with recombinant protein-CCL17 (Rh-CCL17) treated HK2 cells by western blotting (Figure S2).

### 3.5. Increased Expression of CCL17 and EMT-Related Proteins in Obstructed Kidneys

**Figure 5:**
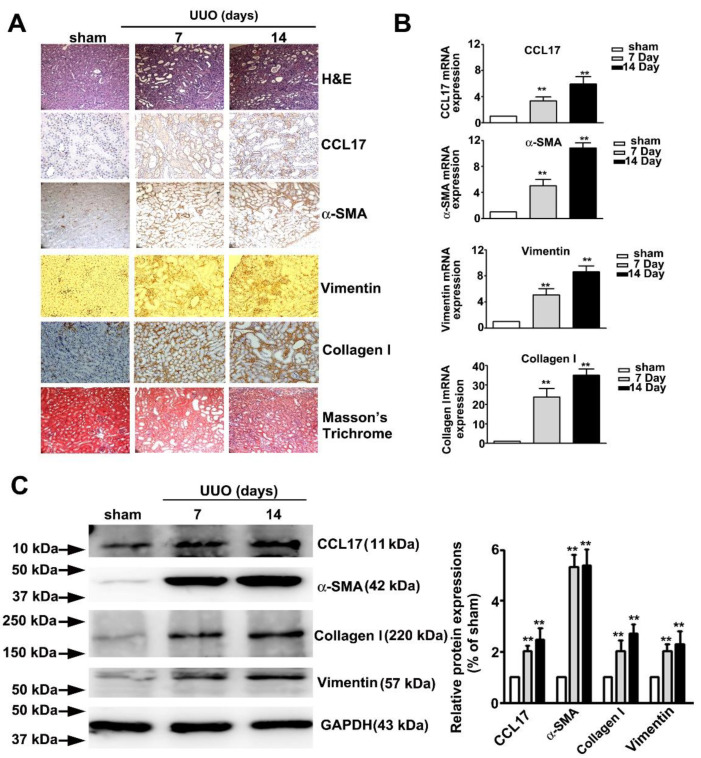
Expression of CCL17 and the morphological transformation of renal tissues in UUO mice. (**A**) Kidney tissue of C57BL/6 mice with unilateral ureteral obstruction (UUO) for 7 and 14 days visualized by hematoxylin and eosin (H&E) stain, immunohistochemical stain (CCL-17, α-SMA, and vimentin), and Masson’s Trichrome staining; (**B**) qRTPCR and (**C**) western blot of mRNA and protein expressions of CCL17, collagen I, α-SMA, and vimentin in sham surgery and UUO mice. GAPDH was used to normalize mRNA loading. Results are expressed as the mean ± SD of at least three independent experiments. ** *p* < 0.01 compared with sham surgery group.

Figure 5 showed the time-dependent morphological features of hematoxylin and eosin (H&E), immunohistochemical (IHC), and Masson tissue staining of UUO mice renal tissues. We observed a marked time-dependent increase in interstitial fibrosis and renal tubule dilatation (upper panel; H&E stain), as well as markedly increased expression of CCL17, α-SMA, vimentin, collagen I (middle panel, IHC statin), and interstitial collagen fibers (lower panel, Masson’s Trichrome stain); consistent with the qRT-PCR results (Figure 5B) and western blotting (Figure 5C). These results suggested that CCL17 played a role in modulating the EMT to promote fibrogenesis in UUO mice.

## 4. Discussion

**Figure 6:**
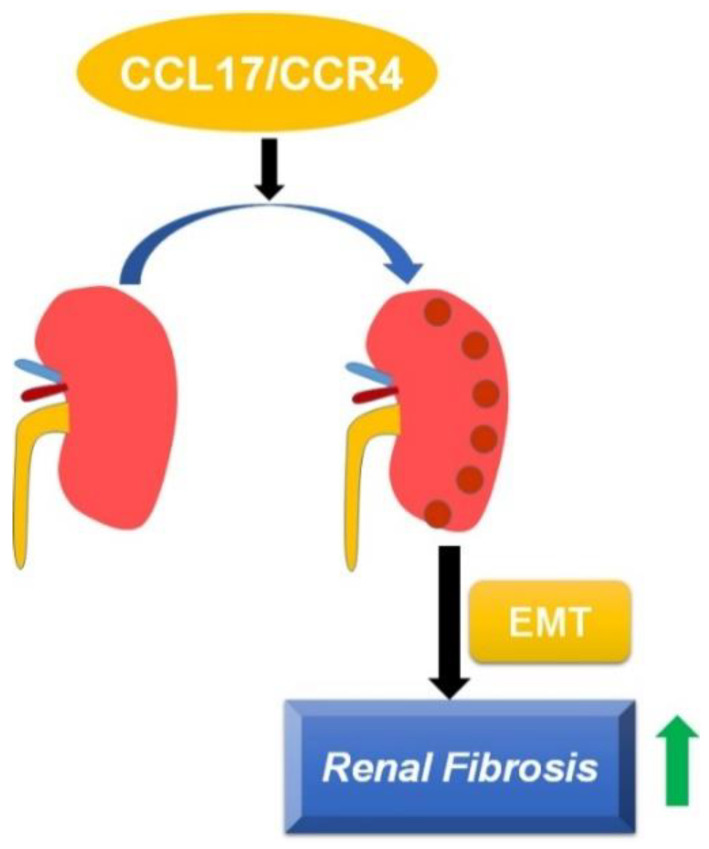
Schematic of CCL17-mediated EMT and fibrotic yields, followed by promoting the development and progression of renal fibrosis.

The involvement of CCL17 in modulating the progression of diseases such as idiopathic pulmonary fibrosis has been reported, but its role in the pathogenesis of CKD remains to be elucidated. In this study, we found that: (1) the CCL17 expression level was negatively associated with renal function in CKD patients; (2) CCL17 overexpression induced phenotype changes in HK2 cells by increasing the expression of α-SMA, collagen, and vimentin in vitro; (3) UUO mice exhibited increased renal interstitial fibrosis, tubular dilatation, and the overexpression of CCL17, α-SMA, collagen I and vimentin in a time-dependent manner. Therefore, CCL17 might be used as a novel biomarker to predict the development of advanced CKD (Figure 6).

Literature studies showed that EMT plays a role in the development and progression of interstitial fibrosis in vitro and in the mouse model. When normal tubular cells were damaged and lost their function, they released paracrine signals to the renal interstitium and affected their microenvironment. During these periods, cells secreted cytokines and chemokines, probably recruited macrophages to the stroma, which lead to the destruction of the renal epithelial cells, the activation of the EMT, and the promotion of fibrogenesis, which were all markers of renal fibrosis [24,25]. The altered phenotype of these cells included upregulated expression of α-SMA and vimentin, along with a dysregulated expression of E- and N-cadherin [4]. Key players in this process included infiltrating leukocytes, interstitial fibroblasts, and myofibroblasts [26]. Evidence has shown that progressive worsening of renal function is associated with α-SMA positive interstitial myofibroblasts in diabetic and membranous nephropathy [5,6]. Studies have shown that substances that block the EMT by mediating inflammation, oxidative stress, and apoptosis in renal tissues can alleviate EMT-induced fibrogenesis [27,28,29]. Consistent with these studies, we observed that CCL17 modulated the phenotypic transformation of renal tubular epithelial cells to induce renal fibrosis as evidenced by the increased expression of α-SMA, vimentin, and collagen I.

Evidence was accumulated that chemokines and their receptors had a strong relationship with poor clinical outcomes in different tumor progression [30], such as that CCR6 and CXCL16/CXCR6 were involved in regulation proliferation, metastasis, and EMT in ESCC and Gastric cancer tumorigenesis [31,32]. Dr Zhong et al. suggested that radiation induced lung toxicity via upregulation of CCL2, CCL5, and CCR4 expression [33]. CCL20/CCR6 induced gastric cancer EMT through the activation of the AKT pathway in response to CrkL [34]; similar results were found with CCL21/CCR7 axis activated JAK2/STAT3 signaling pathways in promoting stemness of OSCC EMT progression [35]. Chemokines and their receptors were chemo-attractants for specific subsets of inflammatory cells, and accumulating evidence indicated that they play important roles in a variety of pathological processes, including renal fibrogenesis and cancer progression [36]. Dr. Zhu found that the condition medium of M2 or CCL17 significantly promoted the proliferation and metastasis of MHCC97L cells through TGF-β1 and Wnt/β-catenin signaling and increased the xenograft MHCC97L tumors in vivo [37]. Studies in UUO mice report significantly increased the expression of CXCL16 in renal tubular epithelial cells and the expression of CXCL16 receptors in circulating fibroblast precursors [38,39]. Moreover, CXCL16 deficiency had been shown to impair the aggregation and myofibroblast production of bone-marrow derived fibroblasts in the kidney and the development of renal fibrosis, while targeted deletion of CXCL16 inhibited myofibroblast activation, reduced collagen deposition, and suppressed collagen I and fibronectin expression [39]. Similar to CXCR6, previous studies have shown that CCL2/CCR2 played a key role in renal damage, especially in the renal tubule interstitium [40,41]. Genetically deficient or medically antagonized CCR2 led to decreased macrophage infiltration and renal fibrosis, as well as decreased expression of CCL2, type I collagen, and TGF-β expression [42]; another study showed that CCR2 knockout mice accumulated fewer bone-marrow derived myofibroblasts and a lower expression of α-SMA and FSP-1 in obstructed kidneys [11]. These findings showed that chemokines and their receptors played an important role in the recruitment of bone-marrow derived fibroblast into kidneys, contributing to renal fibrosis. Consistent with these studies, our study showed that over-expression of CCL17 promoted renal fibrosis progression by stimulating the EMT with an accumulation of collagen. In addition, we found that CCL17 was significantly elevated in CKD patients and that CCL17 expression levels correlated negatively with renal function. CCL17 may be a novel biomarker of advanced CKD (eGFR calculated by MDRD or CKD-EPI formula), with an optimal cut-off value of 415.3 ng/mL and 413.2 ng/mL, respectively. Our study provided a hypothesis that CCL17 had pro-fibrogenetic effects by demonstrating that its overexpression increased phenotype transformation and motility in renal tubule epithelial cells and ECM accumulation in vitro and in vivo, as well as being clinically useful as a novel biomarker that correlated negatively with renal function in CKD patients. Recently, The Food and Drug Administration promoted further studies of CKD273 as diagnosis and risk method for the prediction in CKD progression [43] and high level of CKD273 in early CKD patients [44]. In future studies, we should be analyzing the relationship between CCL17 and CKD273, which will focus on predicting biomarkers of all CKD patients and would require further investigation.

CCL17 was involved in the process of pulmonary fibrosis through CCR4-positive alveolar lymphocytes and macrophages [13,14], and the level of CCL17 correlated positively with disease activity and was used as a biomarker for monitoring the severity of atopic dermatitis [15]. Humans and mice with pulmonary fibrosis were reported to have significantly elevated CCL17 and CCR4 expression in lung tissues, and the severity of pulmonary fibrosis decreased after neutralization of CCL17 [45]. Blocking the expression of CD248, which was upregulated in CKD fibroblasts, attenuated the accumulation of macrophages and downregulated transcription of the CCL17 cytokine gene in macrophages isolated from UUO kidneys. Moreover, CCL17 was upregulated in macrophages induced to phenotypically switch by a hypochlorite injury and increased the migration and type I collagen production in sub-mesothelial fibroblasts; peritoneal adhesion, thickening of the fibrotic peritoneum, and accumulation of myofibroblasts that were attenuated by treatment with an anti-CCL17 antibody [16]. Renal allograft biopsies exhibited a significant progressive upregulation of CCL17 genes from the control group to subclinical inflammation to clinical inflammation, and CCL17 expression correlated clinically with worsening of graft function during a 2-year follow up [17]. Future studies may be focused on developing a CCL17 knockout mice and then observing the influences clinically and pathologically with the UUO model. Furthermore, we would keep the follow-up of these patients longitudinally to examine the role CCL17 in the long-term outcomes of these patients; TGF-β1 inhibited TNF-α and IFN-γ-induced CCL17 production via Smad2/3 in human HaCaT cells [46]. Another study, adenylyl cyclase-cAMP (cAMP) or casuarinin inhibiting TNF-α/IFN-γ-induced CCL17 expression through blocking of p38/NF-κB expression against human HaCaT cell line [47,48]. The precise molecular mechanisms of TGF-β1/Smad/MAPKs/NF-κB was a signaling pathway as a regulation for CCL17 expression in renal fibrosis in vitro and in vivo which is currently under investigation.

In our study we provided a hypothesis that CCL17 has pro-fibrogenetic effects by demonstrating that its overexpression increases of phenotype transformation and motility in renal tubule epithelial cells and ECM accumulation in vitro and in vivo as well as being clinically useful as a novel biomarker that correlates negatively with renal function in CKD patients.

## 5. Conclusions

This study highlights the potential use of CCL17 as a biomarker to predict the development of advanced CKD and as a promising target for treating CKD patients. Further research is needed to gain an in-depth understanding of the mechanism underlying the promotion of renal fibrosis progression by CCL17.

## Figures and Tables

**Table 1:** Baseline characteristic of eGFR_CKD-EPI_ for normal control, early CKD and advanced CKD patients.

		Early CKD			Advanced CKD		
CKD Stage	Normal	1	2	3a	3b	4	5
**Number**	65	12	38	41	52	41	50
**Age (year)**	50.17 ± 10.70	52.42 ± 12.43	64.29 ± 10.28	71.22 ± 9.73	72.02 ± 10.52	75.49 ± 9.59	72.22 ± 13.29
**Female/Male**	35/30	4/8	11/27	13/28	19/33	20/21	27/23
**BUN (mg/dL)**	9.09 ± 2.73	10.91 ± 3.34	15.76 ± 5.35	19.34 ± 5.97	25.00 ± 7.16	37.22 ± 112.53	68.60 ± 24.15
**Cre (mg/dL)**	0.75 ± 0.13	0.77 ± 0.12	1.03 ± 0.18	1.30 ± 0.20	1.69 ± 0.29	2.40 ± 0.46	6.37 ± 2.66
**eGFR_CKD-EPI_ (mL/min)**	101.58 ± 7.42	99.98 ± 10.67	71.43 ± 8.09	51.26 ± 4.40	36.54 ± 4.34	22.95 ± 4.26	8.27 ± 3.44
**CCL17 (ng/mL)**	250.01 ± 138.08	275.26 ± 160.47	333.63 ± 356.98	408.15 ± 291.38	431.22 ± 330.61	485.14 ± 407.62	638.84 ± 429.59
	238.10 (149.56–321.72)	248.05 (174.65–328.24)	258.80 (123.20–403.20)	355.50 (205.05–590.53)	323.85 (203.60–554.48)	373.68 (195.85–633.03)	486.95 (348.60–808.60)
**CCL22 (ng/mL)**	519.03 ± 217.67	465.55 ± 235.42	430.75 ± 241.08	475.77 ± 239.78	584.84 ± 387.71	590.36 ± 572.84	457.72 ± 275.97
	467.90 (368.60–634.10)	427.90 (351.20–482.45)	389.40 (252.20–507.60)	475.50 (246.98–595.28)	509.20 (271.60–841.85)	449.30 (209.75–737.10)	380.30 (221.10–626.00)

Abbreviation: BUN, blood urea nitrogen; Cre, creatinine; eGFR, estimated glomerular filtration rate; early CKD indicated CKD stage 1 to 3a; advanced CKD indicated CKD stage 3b to 5; CKD-EPI, Chronic kidney disease Epidemiology Collaboration.

## Data Availability

The authors will freely release all data underlying the published paper upon direct request to the corresponding author.

## Supplementary Materials

The following are available online at https://www.mdpi.com/article/10.3390/cells10123345/s1, Figure S1: Upregulation of CCL17 expression with loss of renal function, Figure S2: Rh-CCL17 induce the fibrotic- and EMT-related proteins in human HK2 cells; Table S1: Baseline characteristic of eGFR_CKD-EPI_ for normal control, early CKD and advanced CKD patients.
