# Supplementary material for: C-C Motif Chemokine Ligand-17 as a Novel Biomarker and Regulator of Epithelial Mesenchymal Transition in Renal Fibrogenesis
Source: Cells. 2021 Nov 29;10(12):3345. doi: 10.3390/cells10123345 (PMC8699042; doi:10.3390/cells10123345)
Supplement: Supplementary file 1 [file cells-10-03345-s001.zip › cells-1360208-supplementary.pdf]

C-C Motif Chemokine Ligand-17 as a Novel Biomarker and Regulator of Epithelial Mesenchymal Transition in Renal Fibrogenesis

Yi-Hsien Hsieh, Wen-Chien Wang, Tung-Wei Hung, Chu-Che Lee and Jen-Pi Tsai

Supplementary Figure 1

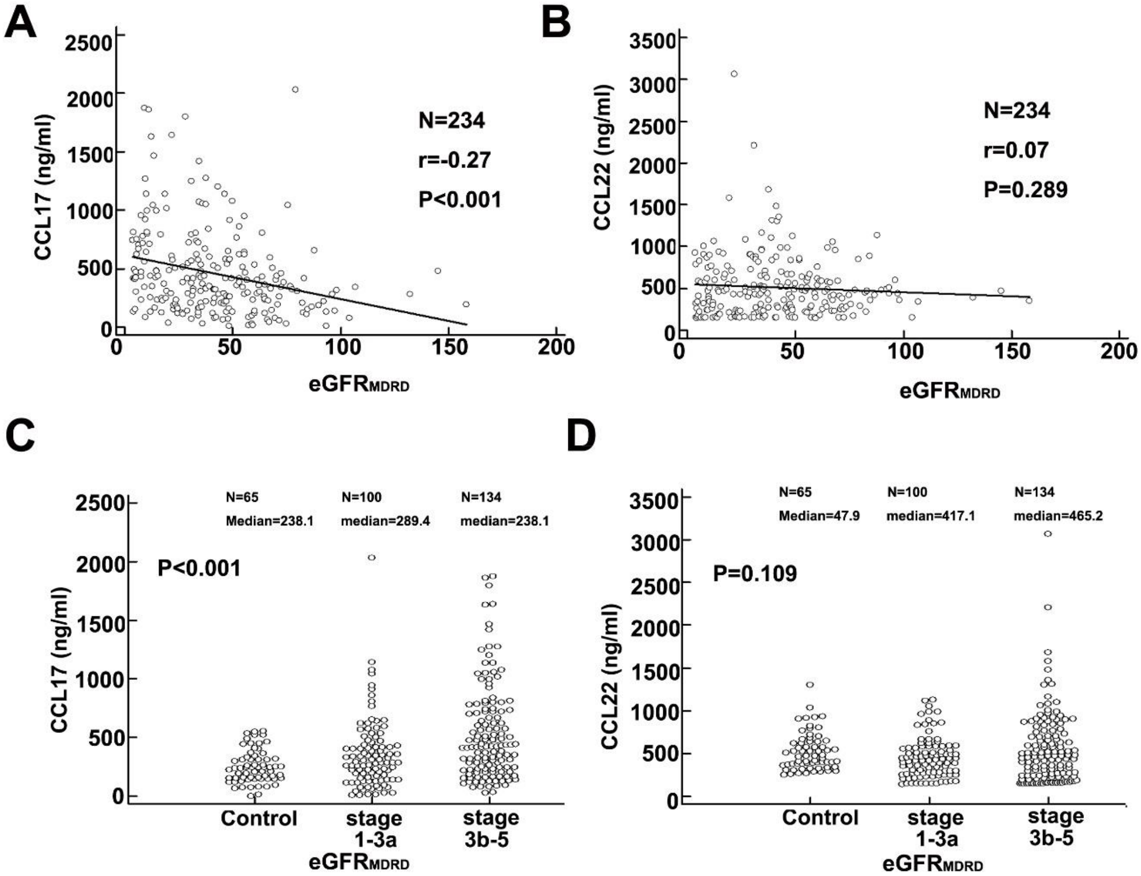

Supplementary Figure S1. Upregulation of CCL17 expression with loss of renal function**.** Correlation analysis of CCL17 (**A**) or CCL22 (**B**) with $eGFR_{MDRD}$ for all CKD patients. Comparison of mean serum values of CCL17 (**C**) and CCL22 (**D**) between the normal population (Control), CKD stage 1–3a and stage 3b–5 with $eGFR_{MDRD}$ for all CKD patients.

Supplementary Figure 2

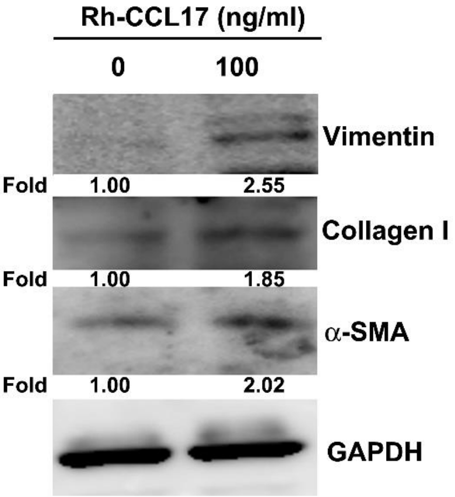

Supplementary Figure S2. Rh-CCL17 induce the fibrotic- and EMT-related proteins in human HK2 cells. Rh-CCL17 treated with or without Rh-CCL17 (100 ng/mL) for 48 h and extracted total HK2 cell lysates to determine the protein expression of CCL17, collagen I, α-SMA, and vimentin. GAPDH was used to normalize protein loading.

Supplementary Table 1

Table S1. Baseline characteristic of $eGFR_{MDRD}$ for normal population and CKD patients.

		Early CKD			Advanced CKD		
Number	65	11	45	44	53	35	46
Age (year)	50.17±10.70	60.91±12.36	64.02±12.14	71.68±10.77	72.60±10.77	74.03±9.17	71.72±13.73
Female/Male	35/30	4/7	14/31	13/31	19/34	18/17	26/20
BUN (mg/dl)	9.09±2.73	11.45±3.93	15.51±5.00	21.11±7.26	26.11±7.40	41.37±18.30	69.43±22.83
Cre (mg/dl)	0.75±0.13	0.72±0.11	1.04±0.16	1.35±0.19	1.80±0.29	2.65±0.56	6.60±2.65
$eGFR_{MDRD}$ (ml/min)	102.69±10.46	110.77±23.33	71.32±8.28	51.47±4.47	36.39±4.05	22.64±4.39	8.88±3.42
CCL17 (ng/ml)	250.01±138.08	222.15±133.50	344.25±327.77	397.04±345.65	475.48±348.55	444.67±408.68	671.90±431.10
	238.10 (149.56-321.72)	218 (128.55-304.73)	300.30 (148.15-405.45)	293.30 (175.83-578.12)	368.30 (206.34-631.46)	285.27 (171.48-535.12)	590.00 (410.90-809.67)
CCL22 (ng/ml)	519.03±217.67	410.06±116.44	4565.65±247.88	437.25±243.61	633.40±440.78	553.24±544.87	463.60±282.65

Abbreviation: BUN, blood urea nitrogen; Cre, creatinine; eGFR, estimated glomerular filtration rate; early CKD indicated CKD stage 1 to 3a; advanced CKD indicated CKD stage 3b to 5; MDRD, Modification of Diet in Renal Disease (MDRD)
